# Sickle Cell Disease: From Ancient Origins to Modern Breakthroughs in Gene Therapy

**DOI:** 10.3390/biomedicines14071649

**Published:** 2026-07-22

**Authors:** Bawo Ikolo, Mathew Oyelami, Odinaka Mgbeke, Kwami Jones, Shellon Thomas, Felicia Ikolo

**Affiliations:** 1 Department of Biology, Ecology & Conservation, School of Arts and Sciences, St. George’s University, True Blue, St. George’s, Grenada; tikolo@sgu.edu; 2 Department of Pathology, School of Medicine, St. George’s University, True Blue, St. George’s, Grenada; moyelami@sgu.edu; 3 Department of Clinical Skills, School of Medicine, St. George’s University, True Blue, St. George’s, Grenada; omgbeke@sgu.edu; 4 Sickle Cell Association of Grenada (SCAG), Kirani James Boulevard, St. George’s, True Blue, St. George’s, Grenada; 5 Department of Biochemistry, School of Medicine, St. George’s University, True Blue, St. George’s, Grenada; kjones@sgu.edu (K.J.); sthoma10@sgu.edu (S.T.)

**Keywords:** sickle cell disease, hemoglobin S, CRISPR/Cas9, gene therapy, vaso-occlusive crisis, hydroxyurea, hematopoietic stem cell transplantation, fetal hemoglobin, hemoglobinopathy, rare disease

## Abstract

Sickle Cell Disease (SCD) is a hereditary hemoglobinopathy arising from a single-nucleotide transversion (GAG → GTG) at codon six of the HBB gene on chromosome 11, substituting glutamic acid with valine in the β-globin chain and producing hemoglobin S (HbS). Under hypoxic conditions, HbS polymerizes and distorts erythrocytes into the characteristic sickle shape, initiating a cascade of vaso-occlusion, chronic hemolytic anemia, and progressive multi-organ damage that defines the clinical burden of this disease. Although SCD has ancient origins in sub-Saharan Africa, the Indian subcontinent, the Middle East, and the Mediterranean, regions where it conferred heterozygous resistance to malaria, the ease of human migration has long since made it a global health concern, affecting an estimated 300,000–400,000 newborns annually. Advances in molecular and genomic research have deepened our understanding of SCD pathophysiology, revealing the central contributions of hemoglobin polymerization, oxidative stress, endothelial inflammation, and nitric oxide depletion to disease progression. Current management rests on supportive pharmacological interventions, including hydroxyurea, chronic transfusion therapy, L-glutamine, and multimodal pain management, complemented by lifestyle modifications. Curative approaches have advanced substantially: hematopoietic stem cell transplantation (HSCT) remains the established standard of cure, while the regulatory approvals in late 2023 of the CRISPR/Cas9-based exagamglogene autotemcel (Casgevy) and the lentiviral vector-based lovotibeglogene autotemcel (Lyfgenia) represent the most transformative development in the history of SCD therapeutics. This review traces the disease from its ancient origins and molecular characterization through to its clinical manifestations, inheritance patterns, screening strategies, and the full spectrum of current and emerging therapies. Persistent challenges, prohibitive treatment costs, healthcare inequities, the ethical dimensions of genome editing, and the urgent need for long-term safety data, are examined critically, with a view to informing the research and policy agenda that must accompany these remarkable scientific advances.

## 1. Introduction

Sickle Cell Disease is, by any measure, one of the most consequential inherited disorders known to medicine. It affects millions of individuals worldwide, with the greatest burden borne by populations in sub-Saharan Africa, where more than 75% of affected births occur annually, though no region is now untouched [1,2]. The irony embedded in this disease’s global distribution is not lost on those who study it closely: SCD endures in part because the heterozygous carrier state, sickle cell trait, confers meaningful resistance to *Plasmodium falciparum* malaria, meaning that natural selection has, over millennia, preserved a mutation that, in homozygous form, causes profound suffering. With human migration now spanning every continent, what was once geographically concentrated is now a diagnosis encountered worldwide.

At its molecular core, SCD results from a single-nucleotide transversion (GAG → GTG) at codon six of the HBB gene, replacing glutamic acid with valine in the β-globin chain and yielding hemoglobin S (HbS) [3,4]. It is worth remembering that when Linus Pauling and his colleagues characterized SCA as the first “molecular disease” in 1949, they were doing something scientifically radical, demonstrating that a single amino acid difference in a protein could explain an entire disease [4]. Vernon Ingram’s identification of the precise substitution in 1957 then gave that conceptual breakthrough its molecular precision [3]. Those two contributions, separated by less than a decade, fundamentally changed how medicine thinks about inherited disease. Under conditions of reduced oxygen tension, HbS polymerizes into rigid, elongated fibers that distort erythrocytes into the sickle shape, impairing their deformability, reducing their lifespan to as little as ten days, and precipitating the cascade of vaso-occlusion, hemolysis, ischemia–reperfusion injury, and sterile inflammation that makes SCD, in the words of those who live with it, relentless [5,6].

Despite the decades of research that have followed Pauling and Ingram, pharmacological management remained limited for most of the twentieth century. Hydroxyurea, first approved for adults with SCD in 1998 and later extended to children, remains the most widely used disease-modifying agent; alongside chronic transfusion therapy, analgesics, and infection prophylaxis, it has improved survival and quality of life without constituting a cure [7,8]. The landscape shifted decisively in late 2023 when both the United States Food and Drug Administration and the UK Medicines and Healthcare products Regulatory Agency approved two gene-based therapies for SCD, Casgevy (exagamglogene autotemcel), a CRISPR/Cas9-based genome editor, and Lyfgenia (lovotibeglogene autotemcel), a lentiviral gene addition therapy [9,10]. These approvals, coming seventy-four years after Pauling first described SCD as a molecular disease, mark a watershed moment. A transformative therapy, it might now be said, is finally in sight, though for the majority of the world’s SCD population, the question of whether it will be universally accessible remains very much open.

This review examines SCD comprehensively, from its historical and cultural contexts through the molecular and genetic foundations of disease, its clinical spectrum, inheritance patterns, screening strategies, and the evolving therapeutic landscape from supportive care to curative gene-based interventions. The ethical, economic, and social obstacles that stand between scientific breakthrough and equitable patient benefit receive deliberate attention, because for a disease that has been known, in its clinical essence, since at least the early twentieth century, the gap between what is scientifically possible and what is practically accessible to affected individuals remains one of the most pressing failures of global health policy.

## 2. Historical and Cultural Perspectives

SCD is older than its molecular characterization by a very long margin. Molecular evidence for the presence of the HbS mutation in antiquity comes from studies applying the amplification refractory mutation system (ARMS) to predynastic Egyptian skeletal remains, placing the sickle cell gene in northeastern Africa thousands of years before modern medicine had any framework for understanding it [11]. The first formally documented clinical case from Egypt was reported in 1951, in a patient and her father, a finding that underscored the hereditary character of what had long been observed but poorly understood [12].

Long before its biomedical characterization, SCD was woven into the cultural and spiritual life of West African communities. The Yoruba term Abiku and the Igbo Ogbanje both described what was effectively SCD in clinical terms, a cycle of birth, recurrent severe childhood illness, and premature death, framed through a belief that the afflicted child was a spirit destined to return repeatedly to the ancestral world [13]. In Ghana, the condition carries the name nsaa, meaning a spiritual affliction, and in many West African communities, children with this pattern of illness were believed to be under malevolent supernatural influence [14]. These cultural narratives were not merely folk misunderstanding; they were coherent attempts to explain a disease whose inheritance pattern, appearing unpredictably, striking some siblings and sparing others, genuinely resisted simple natural explanation before genetics. The persistence of this stigma in contemporary Africa, where many families still encounter reluctance to disclose SCD status for fear of social consequences, is documented and remains clinically relevant [15].

The formal clinical history of SCD in Western medicine begins with Dr. James B. Herrick’s 1910 case report describing “peculiar elongated and sickle-shaped red blood corpuscles” in a patient presenting with severe anemia [16]. That patient was Walter Clement Noel, a young Grenadian dental student who left his home island in September 1904 to pursue his dental education at the Chicago College of Dental Surgery in the United States [17]. It is important to understand that Noel did not travel in comfort. He sailed from Barbados to New York aboard the SS Cearense and, during that week-long sea voyage northward to New York, developed a leg ulcer, a common and painful acute complication of SCD [17]. That leg ulcer was not an isolated event; it was one manifestation of a disease that shadowed him at every stage of his journey. Throughout his years of dental study in Chicago, Noel was repeatedly hospitalized: for bronchitis, for musculoskeletal pain crises, and for what are now recognized as episodes of acute chest syndrome, the leading acute cause of death in SCD patients [17]. Noel studied, sat examinations, and built a professional life while living inside a body in almost constant revolt. And yet he completed his dental degree, his blood sample was analyzed in the clinical investigation that would place SCD permanently in the medical record, and he returned to Grenada to serve his community as a dentist until his death in May 1916 at the age of thirty-two [17]. As Savitt and Goldberg documented in their detailed reconstruction of the case, it was in fact Herrick’s intern, Ernest E. Irons, who first performed the blood work and observed the unusual cells before bringing them to Herrick’s attention, a reminder that this foundational discovery rested on more hands than the single name it now carries [18].

Noel’s contribution to medical science was, for over a century, acknowledged principally by researchers, his name appearing in the foundational literature of molecular medicine, but formally unrecognized by the state. Following sustained advocacy of the Sickle Cell Association of Grenada (SCAG), that changed on 7 February 2026, when the Government of Grenada, on the occasion of its 52nd Independence Day, conferred upon Walter Clement Noel the Knight Grand Cross of the Most Distinguished Order of the Nation posthumously, in recognition of his distinguished and outstanding service to Grenada. The award was proclaimed by Governor-General Dame Cecile Ellen Fleurette La Grenade under Statutory Rules and Orders No. 3 of 2026, signed 4 March 2026, with effect from Independence Day [19]. The formal investiture ceremony followed in March 2026. Under the National Honors and Awards Act, Chapter 204A, Section 10, posthumous awards are explicitly provided for, and the citation—“for distinguished and outstanding service to Grenada” [19]—reflects the full weight of what Noel gave: his blood sample and body as a subject of research, his determination as a student, his professional service as a dentist, and ultimately his place in the history of a disease that has shaped the lives of hundreds of millions of people across the world. That Grenada chose to confer its national knighthood on a man who lived and died over a century ago, who did so carrying the very disease that this review addresses, is a recognition not only of one individual but also of the essential role that patients, not only scientists, play in the making of medical knowledge. Sir Walter Clement Noel, KGCN, deserves to stand in the historical record of SCD alongside Herrick, Pauling, and Ingram, and the Government of Grenada has now formally placed him there.

Beyond Africa, the distribution of the HbS gene in India, where Sickle Cell Disease is prevalent, particularly among tribal populations, and in Mediterranean populations, especially Greece and Italy, where historical accounts of unexplained episodic pain are now considered consistent with SCD, reflects the same underlying logic: wherever *Plasmodium falciparum* malaria was endemic, the sickle cell mutation was selectively favored in heterozygous carriers [20,21]. Understanding this historical and geographical context is not merely academic. It explains the disease’s global distribution and informs the populations in whom screening should be prioritized [15].

## 3. Genetic and Molecular Basis of SCD

Although SCD is classified as a monogenic disorder, and in that sense deceptively simple, its pathophysiology is anything but. The disease originates from a single-nucleotide transversion (GAG → GTG) at codon six of the HBB gene on chromosome 11, substituting the polar, hydrophilic glutamic acid residue with the nonpolar, hydrophobic valine at position 6 of the β-globin polypeptide chain [3]. That substitution, the replacement of one amino acid out of the 146 that constitute the β-globin chain, is sufficient to produce HbS, which, unlike normal adult hemoglobin (HbA), undergoes oxygen concentration-dependent polymerization upon deoxygenation, forming long, insoluble fibers that distort erythrocytes into the sickle shape and reduce their lifespan from the normal 120 days to as few as ten to twenty days [4,5].

The downstream consequences of HbS polymerization are multiple and interconnected. Sickled erythrocytes exhibit markedly increased adhesiveness to vascular endothelium, impaired rheology, and heightened susceptibility to intravascular hemolysis, producing vaso-occlusion, ischemia–reperfusion injury, and chronic inflammation [6,22]. The hemolysis that accompanies sickling is not merely a passive consequence; it is itself a driver of pathology. Free hemoglobin released from lysed erythrocytes scavenges nitric oxide (NO) with high efficiency, while co-released arginase depletes L-arginine, the substrate for NO synthesis; the resulting NO deficiency promotes vasoconstriction, platelet activation, and endothelial dysfunction, and is closely linked to the elevated risk of pulmonary hypertension in SCD patients [23]. Sickled cells also generate excess reactive oxygen species through Fenton chemistry and NADPH oxidase activation, compounding endothelial inflammation through lipid peroxidation and cytokine release, with circulating levels of TNF-α, IL-1β, and IL-6 elevated even in steady-state disease [24,25].

The severity of SCD is not uniform, and that variability has a molecular explanation. Fetal hemoglobin (HbF; α_2_γ_2_) does not participate in HbS polymer formation, and its persistence into adult life, as occurs in hereditary persistence of fetal hemoglobin (HPFH), is associated with a substantially milder phenotype. The transcriptional repressors BCL11A and MYB are the principal genetic modulators of γ-globin silencing in adult erythropoiesis, making them, as has become abundantly clear in the gene therapy era, the most tractable therapeutic targets in the entire disease [26]. It is not coincidental that both approved gene therapies for SCD (Casgevy and Lyfgenia) exploit this biology, the former by disrupting the BCL11A erythroid enhancer to derepress HbF, and the latter by introducing an anti-sickling β-globin transgene directly [27,28]. SCD encompasses several genotypes beyond classical homozygous HbSS, including HbSC and HbSβ-thalassemia (HbSβ^0^ and HbSβ^+^), all of which share the fundamental pathophysiological consequences of HbS polymerization, though to varying degrees of severity [29].

## 4. Clinical Manifestations

SCD is, in the truest clinical sense, a multisystem disease. Its manifestations are the direct consequence of episodic and chronic microvascular occlusion, hemolytic anemia, and the sustained inflammatory state that characterizes even steady-state disease. No organ system is entirely spared, and it is this breadth of involvement, combined with the unpredictability of acute crises, that makes SCD so profoundly disruptive to the lives of those who carry it. Phenotypic severity varies considerably based on genotype, HbF level, co-inheritance of α-thalassemia, and the quality of healthcare available.

### 4.1. Hematological Manifestations and Vaso-Occlusive Crisis

Chronic hemolytic anemia, with hemoglobin levels typically between 6 and 9 g/dL and compensatory reticulocytosis, is the hematological signature of SCD [7]. Hyperbilirubinemia and jaundice from continuous erythrocyte destruction are common, and patients are in a state of chronic hematopoietic stress that leaves little reserve when additional physiological demands arise. Superimposed on this baseline is the vaso-occlusive crisis (VOC)—the hallmark acute presentation, characterized by intense, episodic pain in the bones, joints, chest, and abdomen arising from microvascular occlusion and resultant tissue ischemia [30,31]. VOC can be triggered by infection, dehydration, cold exposure, or psychological stress, and its management remains predominantly symptomatic, a fact that speaks to how far we still have to go in addressing the acute disease burden even in well-resourced settings.

### 4.2. Neurological, Pulmonary, and Splenic Manifestations

Cerebrovascular disease is among the most feared complications of SCD. Overt ischemic stroke occurs in approximately 11% of patients with HbSS by the age of twenty, while silent cerebral infarctions, detectable only by MRI, affect up to one-third of affected children and carry significant cognitive and academic consequences [32]. Acute chest syndrome (ACS) is defined by a new pulmonary infiltrate with pyrexia, chest pain, cough, dyspnea, and hypoxia; its etiology is multifactorial, encompassing pulmonary fat embolism from necrotic bone marrow, infection, and in situ vascular occlusion, and it frequently requires hospitalization, oxygen therapy, and blood transfusion [33]. Recurrent sickling in the splenic sinusoids leads to progressive infarction and ultimately autosplenectomy, rendering patients functionally asplenic and profoundly susceptible to infections from encapsulated organisms, a vulnerability that is the primary rationale for early penicillin prophylaxis and pneumococcal vaccination [34].

### 4.3. Organ Dysfunction

The renal microvasculature, with its hyperosmotic, hypoxic medullary environment, is particularly susceptible to sickling, producing hyposthenuria, hematuria, proteinuria, and, over time, chronic kidney disease with progressive loss of glomerular filtration [35]. Cardiac involvement is driven by the high-output state imposed by chronic anemia, alongside the vasculopathic consequences of hemolysis-associated NO depletion, leading to left ventricular hypertrophy and pulmonary hypertension, the latter being a significant predictor of mortality [36]. Hepatic complications encompass sickle hepatopathy, cholestasis, and iron overload from chronic transfusion therapy, all of which compound the metabolic burden carried by patients over their lifetimes [37].

### 4.4. Musculoskeletal, Ocular, Dermatological, and Reproductive Manifestations

Avascular necrosis of the femoral and humeral heads, bone infarctions, osteomyelitis (most commonly caused by *Salmonella* species, a finding that remains clinically under-recognized), and chronic bone pain constitute the musculoskeletal burden of SCD [38]. Proliferative sickle retinopathy, arising from peripheral retinal ischemia and neovascularization, carries a risk of vitreous hemorrhage and retinal detachment that demands regular ophthalmological surveillance [39]. Chronic leg ulcers, particularly at the malleoli, affect a substantial proportion of adults with HbSS and are among the most refractory and quality-of-life-impairing complications of the disease [40]. Chronic anemia and elevated metabolic demands impair somatic growth and delay puberty, while priapism, arising from obstructed venous outflow during sickling, represents a significant reproductive health risk for male patients, with risk of erectile dysfunction and fibrosis if episodes go untreated [41,42,43].

That a single amino acid substitution can produce this breadth of clinical devastation is both a tribute to the integrative complexity of human physiology and a reminder of why SCD demands genuinely multidisciplinary care, one that integrates hematology, nephrology, cardiology, neurology, ophthalmology, and reproductive medicine within a framework that centers the patient’s experience of living with a chronic, painful, and unpredictable disease.

### 4.5. Psychological, Psychosocial, and Quality-of-Life Dimensions

Any account of the clinical burden of SCD that stops at the body’s organ systems is incomplete. The disease exacts a substantial psychological and psychosocial toll that can shape daily functioning as profoundly as any single physical complication. Depression and anxiety are considerably more common among individuals living with SCD than in the general population; a systematic review of the adult literature confirmed a high burden of depressive symptoms, while emphasizing that reported prevalence varies widely with the screening instruments and thresholds used [44]. Chronic pain, unpredictable crises, repeated hospitalizations, and the lived experience of disease-related stigma all erode quality of life, and health-related stigma in particular remains, in many communities, a barrier to disclosure and to timely, respectful care [15]. A 2026 consensus report of the National Academies of Sciences, Engineering, and Medicine underscored the cumulative, multidimensional burden of living with SCD, including its mental-health dimensions, and argued for evaluating the disease in terms of whole-person functioning rather than discrete organ pathology [45].

Beyond mood and anxiety disorders, the relationship between SCD and the wider spectrum of psychiatric illness is an area of legitimate and growing interest. Because sickled erythrocytes drive a chronic inflammatory state, with sustained elevation of circulating cytokines such as IL-6 and of C-reactive protein, and because chronic peripheral inflammation has been implicated in the pathophysiology of several major psychiatric disorders, it is biologically plausible that the systemic inflammatory milieu of SCD could intersect with neuropsychiatric vulnerability; emerging work has begun to examine how hematopoietic and cellular processes, including those relevant to transplantation, might influence mental-health phenotypes [46]. In our view, however, the present evidence warrants a deliberately cautious framing. While the inflammatory biology of SCD offers a coherent mechanistic rationale for elevated psychiatric risk, a direct causal link between SCD and specific disorders such as schizophrenia remains unestablished, and the well-documented burden of depression and anxiety in this population is more parsimoniously explained by the combined effects of chronic pain, disability, and sustained psychosocial stress. We therefore regard the inflammation–neuropsychiatric interface as a promising direction for future mechanistic research rather than a settled feature of the disease, and we highlight it here to encourage the rigorous, hypothesis-driven study the question deserves.

## 5. Inheritance Patterns and Genetic Screening

SCD follows a strictly autosomal recessive inheritance pattern. Individuals who are heterozygous for the HbS allele (HbAS) carry sickle cell trait and are, under most circumstances, clinically asymptomatic, though strenuous physical exertion under conditions of dehydration or altitude can precipitate complications even in trait carriers, a point that is not always adequately communicated in clinical settings [47]. Homozygosity (HbSS) or compound heterozygosity (HbSC; HbSβ-thalassemia) gives rise to clinically overt SCD. When both parents carry sickle cell trait, each pregnancy carries a one-in-four probability of producing an affected child, a one-in-two probability of producing a carrier, and a one-in-four probability of producing an unaffected non-carrier, a Mendelian distribution that has profound implications for family planning counseling in high-prevalence communities [6].

Newborn screening represents the single most impactful public health intervention for SCD at the population level. Early diagnosis, before the onset of life-threatening complications such as overwhelming pneumococcal sepsis and splenic sequestration crisis, enables timely initiation of penicillin prophylaxis, vaccination, and parental education—interventions that have transformed infant mortality from SCD in settings where screening programs are well-implemented [48]. Prenatal diagnosis through chorionic villus sampling or amniocentesis provides at-risk couples with molecular information to guide reproductive decisions [49]. Preimplantation genetic diagnosis (PGD), used in conjunction with in vitro fertilization, extends this further by enabling the selection of unaffected embryos prior to uterine transfer, a technology whose availability remains largely confined to high-income settings despite being precisely where the global SCD burden is most concentrated [50]. The legal status of this technology is, however, far from uniform. Preimplantation genetic testing is tightly regulated in much of Europe, where dedicated statutory authorities restrict the conditions under which it may be used; it is essentially unregulated at the federal level in the United States; and it remains restricted, prohibited, or simply unavailable in many other jurisdictions. This regulatory patchwork not only shapes which families can access embryo selection for SCD but also drives cross-border “reproductive tourism” and raises distinct ethical and equity concerns that any global strategy for the disease must confront [51,52].

Population-based carrier screening, with appropriate genetic counseling, is established practice in several high-prevalence countries and has demonstrably reduced SCD incidence where implemented systematically. The critical challenge is extending these programs to the settings, particularly sub-Saharan Africa, where SCD burden is greatest and screening infrastructure is least developed [1,48].

## 6. Current and Emerging Treatment Approaches

The therapeutic history of SCD can be read as a slow progression from symptom management to disease modification to, in the present era, genuine cure, though the distribution of that cure remains profoundly inequitable. The sections that follow address each therapeutic tier in sequence.

### 6.1. Supportive and Pharmacological Therapies

Children with SCD are at high risk for overwhelming infections from encapsulated organisms, primarily *Streptococcus pneumoniae*, because functional asplenia develops early in life as a consequence of recurrent splenic sickling. Twice-daily oral penicillin prophylaxis, initiated in infancy and continued until at least five years of age, was established as life-saving by the landmark PROPS trial [8] and remains a cornerstone of pediatric SCD management. Routine immunization with pneumococcal conjugate and polysaccharide vaccines, meningococcal conjugate vaccine, *Haemophilus influenzae* type b vaccine, and annual influenza vaccination complements penicillin prophylaxis in reducing infection-related morbidity and mortality [53].

Hydroxyurea (hydroxycarbamide) is the most important pharmacological advance in SCD management since penicillin prophylaxis. By stimulating HbF production through nitric oxide-mediated signaling and ribonucleotide reductase inhibition, it reduces the intracellular concentration of HbS available for polymerization and decreases the frequency of VOC, acute chest syndrome episodes, and blood transfusion requirements. It is approved for adults and children aged two years and older, yet remains markedly underutilized globally, particularly in Africa, where the majority of affected individuals live; the landmark REACH trial has since established that hydroxyurea is feasible, safe, and effective for children in exactly these settings, providing a strong evidence base for expanding access across sub-Saharan Africa [7,54]. Chronic erythrocyte transfusion therapy, by diluting the proportion of HbS-containing cells—reduces stroke risk, particularly in children identified by transcranial Doppler ultrasonography as high-risk, though long-term iron overload and alloimmunization remain significant management challenges [55]. Supplemental oxygen addresses the hypoxic trigger of HbS polymerization in acute settings [6,56], while L-glutamine (Endari™), approved by the FDA in 2017, augments erythrocyte NAD^+^ redox potential and was shown in a Phase III trial to reduce the frequency of painful crises and hospitalizations [57]. Folic acid supplementation supports ongoing erythropoiesis given the high cell turnover of hemolytic anemia [58], and adequate hydration, often underemphasized in clinic, remains one of the most accessible and effective strategies for reducing vaso-occlusive episodes [59].

Pain management in VOC remains a significant clinical challenge. A multimodal analgesic approach, combining opioids with NSAIDs and adjunctive agents, is standard practice, but the stigma and under-treatment that SCD patients encounter in emergency settings, where their pain is too often doubted or minimized, constitute a documented and shameful failure of healthcare systems [30,31].

### 6.2. The Role of Lifestyle Modifications in SCD Management

Pharmacological and curative interventions do not exhaust the clinician’s toolkit. Lifestyle modifications, while they cannot substitute for medical therapy, are accessible, low-cost, and carry meaningful impact on the frequency and severity of disease-related complications. Tobacco use compromises oxygen transport and exacerbates endothelial inflammation, directly increasing the risk of VOC and acute chest syndrome; smoking cessation is unambiguously recommended for all patients [60]. Alcohol promotes dehydration and can impair adherence to medication and hydration protocols, both of which matter enormously in SCD [61]. Strenuous exercise under conditions of dehydration, high altitude, or extreme temperature can precipitate acute hypoxia and sickling; this does not mean that patients should avoid physical activity, which carries well-documented cardiovascular and psychological benefits, but that activity should be individualized and accompanied by appropriate hydration and rest [47].

Adequate fluid intake, at least eight to ten glasses of water daily in adults, is one of the simplest and most effective anti-sickling interventions available [59]. Patients should be counseled to avoid hypoxia-inducing environments, including high altitudes and poorly ventilated spaces, and to dress appropriately for cold weather, given that vasospasm is a known precipitant of sickling [6]. A nutritionally balanced diet with adequate folic acid, iron where appropriate, zinc, and antioxidant vitamins supports erythropoiesis and mitigates the oxidative stress burden of the disease [30,58]. These recommendations are not new, but their consistent integration into clinical consultations, particularly in primary care settings where many SCD patients receive much of their routine care, remains incomplete.

### 6.3. Curative Therapies: Hematopoietic Stem Cell Transplantation

Allogeneic hematopoietic stem cell transplantation (HSCT) has been, since the first successful procedure in 1984, the only established cure for SCD. By replacing the patient’s defective hematopoietic stem cell compartment with donor-derived cells capable of producing normal hemoglobin, it can eliminate the disease entirely. Outcomes are best when a human leukocyte antigen (HLA)-matched sibling donor is available; disease-free survival rates exceeding 90% have been reported in pediatric patients transplanted early, before significant end-organ damage has accumulated [62]. The fundamental limitation is donor availability: the majority of patients with SCD do not have an HLA-matched sibling, and graft-versus-host disease (GVHD), graft failure, and transplant-related mortality constrain eligibility, particularly in older patients [63].

Advances in conditioning regimens, particularly reduced-intensity and non-myeloablative protocols, and in haploidentical transplantation using post-transplant cyclophosphamide for GVHD prophylaxis, are expanding eligibility and broadening the donor pool, though success rates in these settings remain lower than in matched sibling transplantation and complication rates remain significant [64]. The development of unrelated donor and cord blood registries continues to improve access, but HSCT remains a procedure concentrated in specialized centers in high-income countries, precisely the inverse of the global SCD burden distribution.

### 6.4. Curative Therapies: Gene Therapy

Gene therapy for SCD, using the patient’s own hematopoietic stem cells, conceptually bypasses the immunological barriers and donor availability constraints of allogeneic HSCT. Two principal strategies have been pursued: gene addition, using lentiviral vectors to deliver a functional anti-sickling β-globin transgene, and gene editing, using CRISPR/Cas9 or related technologies to modify the patient’s own genome.

Lyfgenia (lovotibeglogene autotemcel) employs a self-inactivating lentiviral vector to introduce a modified β-globin gene (encoding the T87Q anti-sickling variant, designated HbA^T87Q^) into autologous hematopoietic stem cells, which are then reinfused after myeloablative conditioning. In the Phase I/II HGB-206 study, 88% of patients in the pivotal Group C cohort achieved complete resolution of severe vaso-occlusive events following treatment [28].

Casgevy (exagamglogene autotemcel) takes a fundamentally different approach, using CRISPR/Cas9 to introduce targeted disruptions in the BCL11A erythroid enhancer within autologous hematopoietic stem cells, abrogating BCL11A’s repression of γ-globin and reactivating HbF production. In the pivotal CLIMB SCD-121 trial, 93.5% of evaluable patients were free from severe VOC for at least twelve consecutive months following treatment [27,65]. Casgevy became the first CRISPR-based therapy approved for any human disease, authorized by the MHRA in November 2023 and by the FDA in December 2023 [9,10]. In 2019, Victoria Gray had become the first SCD patient in the world to receive CRISPR-based gene editing, and her sustained clinical response, freedom from sickle cell crises over years of follow-up, provided the clinical proof of concept that made these approvals possible [66].

Base editing and prime editing technologies, which achieve precise, programmable nucleotide changes without requiring double-stranded DNA breaks, are now under investigation for SCD and may offer improved safety profiles by reducing off-target indels and chromosomal rearrangements [67,68]. Despite the extraordinary promise of these approaches, the requirement for myeloablative conditioning, the complexity of autologous cell product manufacturing, and the current cost structure of these therapies represent obstacles that will need to be resolved before gene therapy can fulfill its potential as a universal cure [29,69].

### 6.5. Recent Regulatory Approvals and Their Significance

The concurrent regulatory approvals of Casgevy and Lyfgenia in late 2023 are, without overstatement, the most significant therapeutic milestone in the history of SCD since hydroxyurea. They validate over two decades of investment in hematopoietic gene therapy, establish for the first time that CRISPR/Cas9-based editing is clinically safe and effective in a hematological disease, and transform the therapeutic horizon for patients who previously had no curative option outside allogeneic HSCT. Whether this transformation is accessible to the populations most affected by SCD is, however, a different and considerably less encouraging question, one addressed directly in Section 7.

## 7. Future Perspectives and Challenges

SCD has been described, diagnosed, and characterized at the molecular level for over a century. Gene therapies capable of curing it now exist. And yet for the overwhelming majority of people living with SCD, predominantly in sub-Saharan Africa, where healthcare expenditure per capita is often below one hundred US dollars per year, the question that one of us raised in an earlier publication [70] remains unanswered: is a universally applicable cure for SCD too slow in coming? The challenges below suggest that the answer is still yes and that scientific progress without equitable access is, at best, an incomplete victory.

The list prices of Casgevy and Lyfgenia in the United States are $2.2 million and $3.1 million per patient, respectively [71]. These figures are not anomalies in the rare disease gene therapy landscape, but for a disease whose burden is overwhelmingly concentrated in low- and middle-income countries, they represent a form of therapeutic inaccessibility so complete as to be morally indefensible [9]. Developing sustainable pricing models, tiered access agreements, and health technology assessment frameworks that acknowledge the economic realities of high-burden, low-income settings is not a technical afterthought; it is a condition of the therapies’ legitimacy as public health interventions.

Long-term safety data remain genuinely limited. Lentiviral vector integration carries a theoretical and, in earlier gene therapy trials, a demonstrated risk of insertional mutagenesis; while no oncogenic events have been reported to date for Lyfgenia, the follow-up duration remains short relative to the decades of life over which these therapies will need to demonstrate safety [28]. CRISPR/Cas9 editing in Casgevy has shown a favorable off-target profile in genomic analyses to date, but the long-term genomic stability of edited hematopoietic stem cell populations requires sustained surveillance [27]. These are not reasons to withhold therapies that have demonstrated compelling clinical efficacy; they are reasons to invest heavily in post-marketing surveillance and to be transparent with patients about the limits of current knowledge.

The ethical dimensions of genome editing in somatic cells, which is what both Casgevy and Lyfgenia involve, are distinct from germline editing, which remains internationally prohibited and whose risks extend to future generations. Somatic gene editing in consenting patients is ethically defensible when the risk-benefit profile is favorable and when informed consent processes are rigorous. The challenge in resource-limited settings is ensuring that consent processes are genuinely informed and that patients are not driven to accept experimental therapies by desperation in the absence of alternatives [72].

Education and awareness remain critical gaps. In many communities where SCD is prevalent, including communities in high-income countries with significant SCD populations, health literacy about the genetic basis of the disease, the importance of carrier screening, and the range of available treatments is demonstrably inadequate. Misinformation, cultural stigma, and inadequate training of healthcare providers all contribute to diagnostic delay and suboptimal management [73,74]. Persons affected by SCD also carry a disproportionate psychosocial burden: chronic pain, depression, social stigma, and diminished educational and occupational attainment are well-documented [75], and the healthcare system’s response to these dimensions of the disease lags far behind its response to the biomedical ones.

The clinical pipeline beyond gene therapy includes several promising agents. Voxelotor, an HbS polymerization inhibitor, crizanlizumab, an anti-P-selectin monoclonal antibody, and mitapivat, a pyruvate kinase activator, have each demonstrated clinical benefit, and combinatorial strategies integrating these mechanisms with HbF inducers may offer synergistic disease modification without the toxicity and complexity of myeloablative conditioning. Looking further forward, in vivo gene editing, delivering gene editors systemically via lipid nanoparticles or viral vectors, without the need for ex vivo hematopoietic stem cell manipulation or hospitalization, represents the approach most likely to make gene-based cure truly accessible to the global SCD population. Achieving it requires continued, sustained investment in delivery systems biology, preclinical safety assessment, and the regulatory science needed to evaluate a new class of living medicines.

## 8. Conclusions

SCD is a disease of extraordinary scientific significance and profound human consequence. It was the first molecular disease to be described, it was among the first in which the causative mutation was precisely identified, and it is now among the first for which CRISPR-based gene editing has received regulatory approval as a therapeutic intervention. The arc from Herrick’s 1910 clinical description [16] through Pauling’s 1949 molecular characterization [4] and Ingram’s 1957 identification of the amino acid substitution [3] to the 2023 approvals of Casgevy and Lyfgenia [9,10] is one of the great narratives of modern molecular medicine, and it is a narrative in which the contributions of researchers from affected populations, including those in the Caribbean, Africa, and the developing world, deserve full recognition alongside those of the major research centers. It is equally a narrative in which the patient deserves formal acknowledgement. The posthumous conferral of the Knight Grand Cross of the Most Distinguished Order of the Nation upon Sir Walter Clement Noel, KGCN, by the Government of Grenada in 2026 is a reminder that medical progress is built not only on the genius of scientists but also on the courage, consent, and endurance of those who live and die with the diseases being studied [19].

And yet SCD remains, for most of the individuals who carry it, a daily burden of pain, uncertainty, and constrained opportunity. Hydroxyurea, an inexpensive and effective disease-modifying agent available for more than two decades, is still markedly underutilized in the regions where SCD is most prevalent. Newborn screening, which can prevent early deaths that have historically characterized the disease in undiagnosed infants, remains inconsistently implemented. And the gene therapies that represent the scientific frontier of SCD treatment are, at current prices and in current healthcare infrastructures, out of reach for the vast majority of patients worldwide.

What is needed, alongside continued investment in scientific research, is a genuine commitment to the public health architecture that converts scientific possibility into clinical reality for all affected individuals. This means universal newborn screening programs, accessible carrier testing with genetic counseling, consistent delivery of established therapies, including hydroxyurea and infection prophylaxis, and policy frameworks, at national and international levels, that ensure new curative therapies do not remain the exclusive province of wealthy healthcare systems. It also means taking seriously the psychosocial burden of SCD and the stigma that remains, in many communities, one of the most persistent barriers to timely diagnosis and appropriate care [15,75].

The cure, as noted above, is finally in sight. Making it universally accessible is the scientific, clinical, ethical, and political challenge of the next generation of SCD researchers and advocates. This review is offered in that spirit.

## Data Availability

No new data were created or analyzed in this study. Data sharing is not applicable to this article.

## Abbreviations

The following abbreviations are used in this manuscript:

ACS	Acute Chest Syndrome
AVN	Avascular Necrosis
CRISPR	Clustered Regularly Interspaced Short Palindromic Repeats
FDA	United States Food and Drug Administration
GVHD	Graft-Versus-Host Disease
HbA	Hemoglobin A (normal adult hemoglobin)
HbF	Fetal Hemoglobin
HbS	Hemoglobin S (sickle hemoglobin)
HBB	β-globin gene
HPFH	Hereditary Persistence of Fetal Hemoglobin
HSC	Hematopoietic Stem Cell
HSCT	Hematopoietic Stem Cell Transplantation
MHRA	Medicines and Healthcare products Regulatory Agency (UK)
NO	Nitric Oxide
PGD	Preimplantation Genetic Diagnosis
RBC	Red Blood Cell
ROS	Reactive Oxygen Species
SCD	Sickle Cell Disease
SCT	Sickle Cell Trait
VOC	Vaso-Occlusive Crisis

## References

[1] Piel F.B., Patil A.P., Howes R.E., Nyangiri O.A., Gething P.W., Williams T.N., Weatherall D.J., Hay S.I. (2010). Global distribution of the sickle cell gene and geographical confirmation of the malaria hypothesis. Nat. Commun..

[2] Egesa W.I., Nakalema G., Waibi W.M., Turyasiima M., Amuje E., Kiconco G., Odoch S., Kumbakulu P.K., Abdirashid S., Asiimwe D. (2022). Sickle Cell Disease in Children and Adolescents: A Review of the Historical, Clinical, and Public Health Perspective of Sub-Saharan Africa and Beyond. Int. J. Pediatr..

[3] Ingram V.M. (1957). Gene mutations in human hemoglobin: The chemical difference between normal and sickle cell hemoglobin. Nature.

[4] Pauling L., Itano H.A., Singer S.J., Wells I.C. (1949). Sickle cell anemia, a molecular disease. Science.

[5] Steinberg M.H. (1999). Management of sickle cell disease. N. Engl. J. Med..

[6] Rees D.C., Williams T.N., Gladwin M.T. (2010). Sickle-cell disease. Lancet.

[7] Ware R.E., de Montalembert M., Tshilolo L., Abboud M.R. (2017). Sickle cell disease. Lancet.

[8] Gaston M.H., Verter J.I., Woods G., Pegelow C., Kelleher J., Presbury G., Zarkowsky H., Vichinsky E., Iyer R., Lobel J.S. (1986). Prophylaxis with oral penicillin in children with sickle cell anemia. N. Engl. J. Med..

[9] FDA (2023). FDA Approves First Gene Therapies to Treat Patients with Sickle Cell Disease.

[10] MHRA (2023). MHRA Authorises World-First Gene Therapy That Aims to Cure Sickle Cell Disease and Transfusion-Dependent β-Thalassaemia.

[11] Marin A., Cerutti N., Massa E.R. (1999). Use of the amplification refractory mutation system (ARMS) in the study of HbS in predynastic Egyptian remains. Boll. Soc. Ital. Biol. Sper..

[12] Abbasy A.S. (1951). Case report: Sickle cell anemia; first case reported from Egypt. Blood.

[13] Nzewi E. (2001). Malevolent ogbanje: Recurrent reincarnation or sickle cell disease?. Soc. Sci. Med..

[14] Konotey-Ahulu F.I.D. (1991). The Sickle Cell Disease Patient.

[15] Anie K.A. (2024). The intersection of sickle cell disease, stigma, and pain in Africa. Hematol. Am. Soc. Hematol. Educ. Program.

[16] Herrick J.B. (1910). Peculiar elongated and sickle-shaped red blood corpuscles in a case of severe anemia. Arch. Intern. Med..

[17] Steensma D.P., Kyle R.A., Shampo M.A. (2010). Walter Clement Noel—First patient described with sickle cell disease. Mayo Clin. Proc..

[18] Savitt T.L., Goldberg M.F. (1989). Herrick’s 1910 case report of sickle cell anemia: The rest of the story. JAMA.

[19] Government of Grenada (2026). National Honors and Awards Proclamation 2026. Statutory Rules and Orders No. 3 of 2026. Signed by Governor-General Dame Cécile Ellen Fleurette La Grenade, 4 March 2026.

[20] Colah R.B., Mukherjee M.B., Martin S., Ghosh K. (2015). Sickle cell disease in tribal populations in India. Indian J. Med. Res..

[21] Pagnier J., Mears J.G., Dunda-Belkhodja O., Schaefer-Rego K.E., Beldjord C., Nagel R.L., Labie D. (1984). Evidence for the multicentric origin of the sickle cell hemoglobin gene in Africa. Proc. Natl. Acad. Sci. USA.

[22] Hebbel R.P., Yamada O., Moldow C.F., Jacob H.S., White J.G., Eaton J.W. (1980). Abnormal adherence of sickle erythrocytes to cultured vascular endothelium: Possible mechanism for microvascular occlusion in sickle cell disease. J. Clin. Investig..

[23] Reiter C.D., Wang X., Tanus-Santos J.E., Hogg N., Cannon R.O., Schechter A.N., Gladwin M.T. (2002). Cell-free hemoglobin limits nitric oxide bioavailability in sickle-cell disease. Nat. Med..

[24] Hebbel R.P., Eaton J.W., Balasingam M., Steinberg M.H. (1982). Spontaneous oxygen radical generation by sickle erythrocytes. J. Clin. Investig..

[25] Belcher J.D., Bryant C.J., Nguyen J., Bowlin P.R., Kielbik M.C., Bischof J.C., Hebbel R.P., Vercellotti G.M. (2003). Transgenic sickle mice have vascular inflammation. Blood.

[26] Bauer D.E., Kamran S.C., Orkin S.H. (2012). Reawakening fetal hemoglobin: Prospects for new therapies for the β-globin disorders. Blood.

[27] Frangoul H., Altshuler D., Cappellini M.D., Chen Y.S., Domm J., Eustace B.K., Foell J., De La Fuente J., Grupp S., Handgretinger R. (2021). CRISPR-Cas9 gene editing for sickle cell disease and β-thalassaemia. N. Engl. J. Med..

[28] Kanter J., Walters M.C., Krishnamurti L., Mapara M.Y., Kwiatkowski J.L., Rifkin-Zenenberg S., Aygun B., Kasow K.A., Pierciey F.J., Bonner M. (2022). Biologic and clinical efficacy of LentiGlobin for sickle cell disease. N. Engl. J. Med..

[29] Tang R., Shan S. (2024). Recent advances in the molecular mechanisms and therapeutic strategies of sickle cell disease. Front. Genet..

[30] Ballas S.K., Gupta K., Adams-Graves P. (2012). Sickle cell pain: A critical reappraisal. Blood.

[31] Yawn B.P., Buchanan G.R., Afenyi-Annan A.N., Ballas S.K., Hassell K.L., James A.H., Jordan L., Lanzkron S.M., Lottenberg R., Savage W.J. (2014). Management of sickle cell disease: Summary of the 2014 evidence-based report by expert panel members. JAMA.

[32] DeBaun M.R., Kirkham F.J. (2016). Central nervous system complications and management in sickle cell disease. Blood.

[33] Vichinsky E.P., Neumayr L.D., Earles A.N., Williams R., Lennette E.T., Dean D., Nickerson B., Orringer E., McKie V., Bellevue R. (2000). Causes and outcomes of the acute chest syndrome in sickle cell disease. N. Engl. J. Med..

[34] Brousse V., Buffet P., Rees D. (2014). The spleen and sickle cell disease: The sick(led) spleen. Br. J. Haematol..

[35] Guasch A., Navarrete J., Nass K., Zayas C.F. (2006). Glomerular involvement in adults with sickle cell hemoglobinopathies: Prevalence and clinical correlates of progressive renal failure. J. Am. Soc. Nephrol..

[36] Gladwin M.T., Barst R.J., Gibbs J.S.R., Hildesheim M., Sachdev V., Nouraie M., Hassell K.L., Little J.A., Schraufnagel D.E., Krishnamurti L. (2014). Risk factors for death in 632 patients with sickle cell disease in the United States and United Kingdom. PLoS ONE.

[37] Banerjee S., Owen C., Chopra S. (2001). Sickle cell hepatopathy. Hepatology.

[38] Almeida A., Roberts I. (2005). Bone involvement in sickle cell disease. Br. J. Haematol..

[39] Emerson G.G., Lutty G.A. (2005). Effects of sickle cell disease on the eye: Clinical features and treatment. Hematol./Oncol. Clin. North Am..

[40] Koshy M., Entsuah R., Koranda A., Kraus A.P., Johnson R., Bellvue R., Zacharias R., Kinney T. (1989). Leg ulcers in patients with sickle cell disease. Blood.

[41] Singhal A., Thomas P., Cook R., Wierenga K., Serjeant G. (1994). Delayed adolescent growth in homozygous sickle cell disease. Arch. Dis. Child..

[42] Mantadakis E., Cavender J.D., Rogers Z.R., Ewalt D.H., Buchanan G.R. (1999). Prevalence of priapism in children and adolescents with sickle cell anemia. J. Pediatr. Hematol./Oncol..

[43] Adeyoju A.B., Olujohungbe A.B., Morris J., Yardumian A., Bareford D., Akenova A., Akinyanju O., Cinkotai K., O’Reilly P.H. (2002). Priapism in sickle-cell disease; incidence, risk factors and complications—An international multicenter study. BJU Int..

[44] Oudin Doglioni D., Chabasseur V., Barbot F., Galactéros F., Gay M.-C. (2021). Depression in adults with sickle cell disease: A systematic review of the methodological issues in assessing prevalence of depression. BMC Psychol..

[45] Spicer C.M., Koop J.I., Volberding P.A., National Academies of Sciences, Engineering, and Medicine (2026). Sickle Cell Disease in Social Security Disability Evaluations.

[46] Tsytsarev V.Y., Volnova A.B., Inyushin M.Y. (2025). Transplanted cells, transferred minds: Can transplanted cells influence mental illness? (A review). Cell Tissue Biol..

[47] Connes P., Hardy-Dessources M.D., Hue O. (2007). Counterpoint: Sickle cell trait should not be considered asymptomatic and as a benign condition during physical activity. J. Appl. Physiol..

[48] Benson J.M., Therrell B.L. (2010). History and current status of newborn screening for hemoglobinopathies. Semin. Perinatol..

[49] Tsaras G., Owusu-Ansah A., Boateng F.O., Amoateng-Adjepong Y. (2009). Complications associated with sickle cell trait: A brief narrative review. Am. J. Med..

[50] Traeger-Synodinos J. (2013). Preimplantation genetic diagnosis, an alternative to conventional prenatal diagnosis of the hemoglobinopathies. Int. J. Lab. Hematol..

[51] Bayefsky M.J. (2016). Comparative preimplantation genetic diagnosis policy in Europe and the USA and its implications for reproductive tourism. Reprod. Biomed. Soc. Online.

[52] Ginoza M.E.C., Isasi R. (2020). Regulating preimplantation genetic testing across the world: A comparison of international policy and ethical perspectives. Cold Spring Harb. Perspect. Med..

[53] Verduzco L.A., Nathan D.G. (2009). Sickle cell disease and stroke. Blood.

[54] Tshilolo L., Tomlinson G., Williams T.N., Santos B., Olupot-Olupot P., Lane A., Aygun B., Stuber S.E., Latham T.S., McGann P.T. (2019). Hydroxyurea for children with sickle cell anemia in sub-Saharan Africa. N. Engl. J. Med..

[55] Adams R.J., Brambilla D. (2005). Discontinuing prophylactic transfusions used to prevent stroke in sickle cell disease. N. Engl. J. Med..

[56] Omoigui S. (2007). The biochemical origin of pain: The origin of all pain is inflammation and the inflammatory response. Part 2 of 3—Inflammatory profile of pain syndromes. Med. Hypotheses.

[57] NIH (2024). L-Glutamine (Endari) for Sickle Cell Disease.

[58] Platt O.S., Brambilla D.J., Rosse W.F., Milner P.F., Castro O., Steinberg M.H., Klug P.P. (1994). Mortality in sickle cell disease: Life expectancy and risk factors for early death. N. Engl. J. Med..

[59] Serjeant G.R. (1997). Sickle-cell disease. Lancet.

[60] Cohen R.T., DeBaun M.R., Blinder M.A., Strunk R.C., Field J.J. (2010). Smoking is associated with an increased risk of acute chest syndrome and pain among adults with sickle cell disease. Blood.

[61] NHS (2025). Sickle Cell Disease—Living with.

[62] Walters M.C., Storb R., Patience M., Leisenring W., Taylor T., Sanders J.E., Buchanan G.E., Rogers Z.R., Dinndorf P., Davies S.C. (2000). Impact of bone marrow transplantation for symptomatic sickle cell disease: An interim report. Blood.

[63] Bernaudin F., Socie G., Kuentz M., Chevret S., Duval M., Bertrand Y., Vannier J.-P., Yakouben K., Thuret I., Bordigoni P. (2007). Long-term results of related myeloablative stem-cell transplantation to cure sickle cell disease. Blood.

[64] Hsieh M.M., Fitzhugh C.D., Tisdale J.F. (2011). Allogeneic hematopoietic stem cell transplantation for sickle cell disease: The time is now. Blood.

[65] Esrick E.B., Lehmann L.E., Biffi A., Achebe M., Brendel C., Ciuculescu M.F., Daley H., MacKinnon B., Morris E., Federico A. (2021). Post-transcriptional genetic silencing of BCL11A to treat sickle cell disease. N. Engl. J. Med..

[66] NPR (2019). A Young Mississippi Woman’s Journey Through A Pioneering Gene-Editing Experiment.

[67] Newby G.A., Yen J.S., Woodard K.J., Mayuranathan T., Lazzarotto C.R., Li Y., Sheppard-Tillman H., Porter S.N., Yao Y., Mayberry K. (2021). Base editing of hematopoietic stem cells rescues sickle cell disease in mice. Nature.

[68] Anzalone A.V., Randolph P.B., Davis J.R., Sousa A.A., Koblan L.W., Levy J.M., Chen P.J., Wilson C., Newby G.A., Raguram A. (2019). Search-and-replace genome editing without double-strand breaks or donor DNA. Nature.

[69] Ballantine J., Tisdale J.F. (2025). Gene therapy for sickle cell disease: Recent advances, clinical trials and future directions. Cytotherapy.

[70] Ikolo B.T., Ikolo F.A. (2018). Universal cure for sickle cell disease is too slow in coming. Int. J. Adv. Sci. Eng. Technol. IJASEAT.

[71] Congressional Budget Office (2024). How Increased Use of Gene Therapy Treatment for Sickle Cell Disease Could Affect the Federal Budget.

[72] Doudna J.A., Charpentier E. (2014). Genome editing. The new frontier of genome engineering with CRISPR-Cas9. Science.

[73] Treadwell M.J., Telfair J., Gibson R.W., Johnson S., Osunkwo I. (2011). Transition from pediatric to adult care in sickle cell disease: Establishing evidence-based practice and directions for research. Am. J. Hematol..

[74] Reich J., Cantrell M.A., Smeltzer S.C. (2023). An integrative review: The evolution of provider knowledge, attitudes, perceptions and perceived barriers to caring for patients with sickle cell disease 1970–now. J. Pediatr. Hematol./Oncol. Nurs..

[75] Essien E.A., Winter-Eteng B.F., Onukogu C.U., Nkangha D.D., Daniel F.M. (2023). Psychosocial challenges of persons with sickle cell anemia: A narrative review. Medicine.

